# Supplementary material for: Streamlining differential exon and 3′ UTR usage with diffUTR
Source: BMC Bioinformatics. 2021 Apr 13;22:189. doi: 10.1186/s12859-021-04114-7 (PMC8045333; doi:10.1186/s12859-021-04114-7)
Supplement: Supplementary file 1 — Additional file 1: Supplementary Figures 1–3. [file 12859_2021_4114_MOESM1_ESM.pdf]

Streamlining differential exon and 3' UTR usage with diffUTR

Stefan Gerber & Pierre-Luc Germain

Supplementary Figures

Supplementary Figure 1

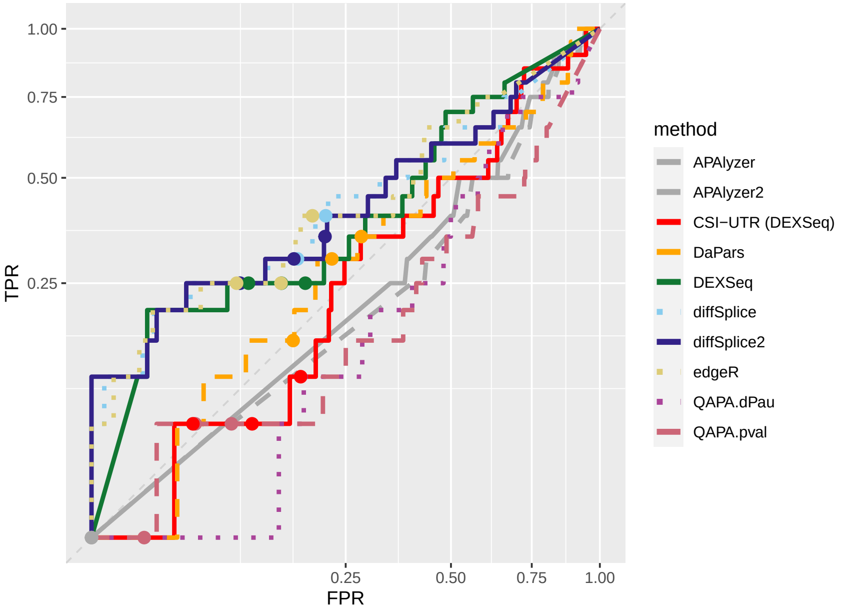

Supplementary Figure 1: Differential UTR accuracy in real data. Receiver-operator characteristic (ROC) curves of differential UTR usage analysis on the LTP dataset, using 3' sequencing to establish the ground truth. The axes are square-root-transformed to improve visibility.

Supplementary Figure 2

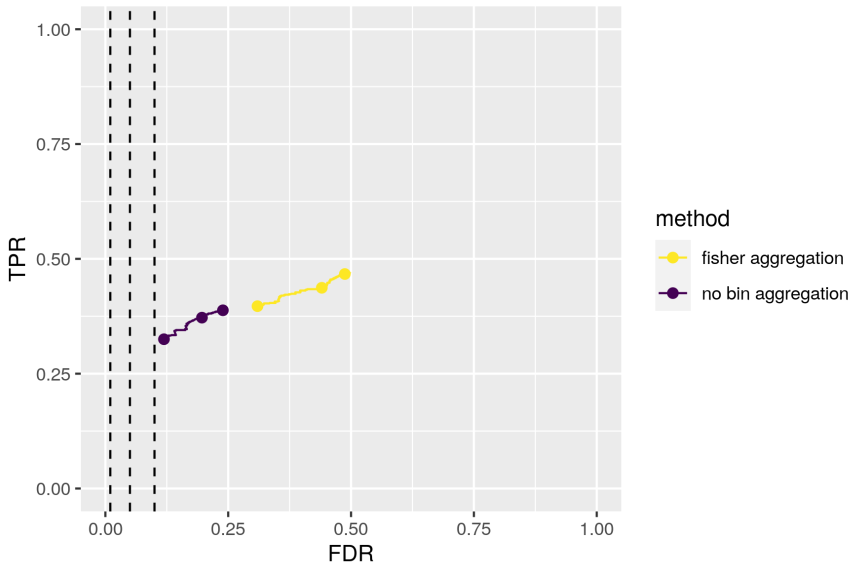

Supplementary Figure 2: Consecutive bin aggregation does not improve diffUTR analysis. True Positive Rate (TPR, i.e. sensitivity) and False Discovery Rate of *diffUTR-diffSplice2* method on the simulated data, when aggregating bins of the same size using Fisher's method. Shown are thresholds of 0.1, 0.05 and 0.01. As expected, p-value aggregation slightly increases sensitivity, but at a greater cost on the already high FDR.

Supplementary Figure 3

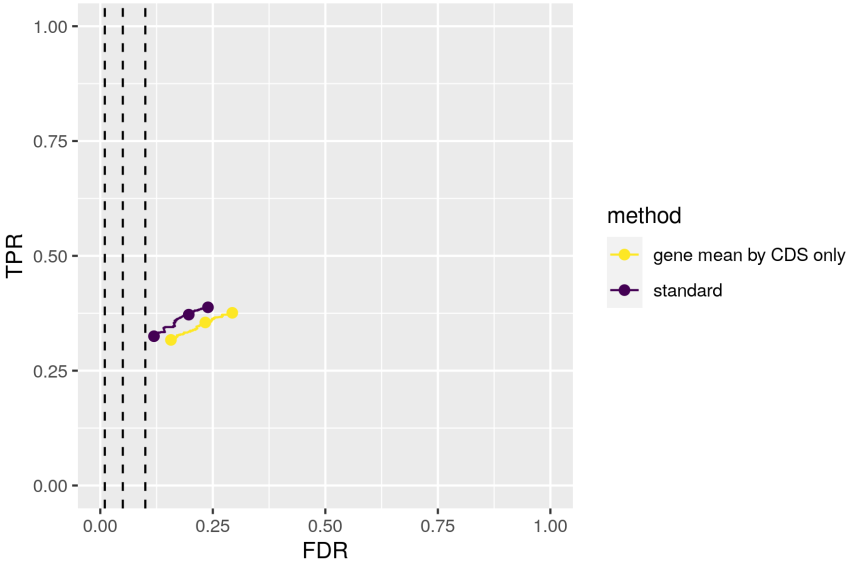

Supplementary Figure 3: Using a CDS-only gene mean does not improve diffUTR analysis. True Positive Rate (TPR, i.e. sensitivity) and False Discovery Rate of *diffUTR-diffSplice2* method on the simulated data, when calculating the gene means based on all bins or only CDS bins. Shown are thresholds of 0.1, 0.05 and 0.01.
